# Protocol for the DREAM Project (Diabetes Research, Education, and Action for Minorities): a randomized trial of a community health worker intervention to improve diabetic management and control among Bangladeshi adults in NYC

**DOI:** 10.1186/1471-2458-14-177

**Published:** 2014-02-18

**Authors:** Nadia Islam, Lindsey Riley, Laura Wyatt, S Darius Tandon, Michael Tanner, Runi Mukherji-Ratnam, Mariano Rey, Chau Trinh-Shevrin

**Affiliations:** 1 Department of Population Health, New York University School of Medicine, 227 East 30th Street, 8 F, New York, NY 10016, USA; 2 Department of Medical Social Sciences, Northwestern University, 750 N Lake Shore, Chicago, IL 60611, USA; 3 Psychology Department, State University of New York, 210 Store Hill Road, Old Westbury, NY 11568, USA

**Keywords:** Health disparities, South Asian Health, Diabetes, CHW, CBPR

## Abstract

**Background:**

New York City (NYC) is currently home to the largest Bangladeshi population in the United States (US) at approximately 62,000 individuals. The high prevalence of Type 2 diabetes mellitus (T2DM) among Bangladeshis has been well documented in Bangladesh, as well as in Canada and the United Kingdom (UK). However, little is known about the diabetes prevalence and management practices of US Bangladeshis. This paper describes the protocol for a Community Health Worker (CHW) intervention to improve diabetic management and control among Bangladeshis with diabetes in NYC.

**Methods/Design:**

For a two-arm, randomized controlled trial (RCT), investigators will recruit a sample of 256 participants, all of whom are 1) of Bangladeshi descent, 2) residing in NYC, 3) diagnosed with T2DM and a recent Hemoglobin A1c (HbA1c) of ≥ 6.5, and 4) between the ages of 21–85. The treatment group receives a six-month CHW-led intervention consisting of five monthly group educational sessions, two one-on-one visits, and follow-up phone calls as needed from a CHW. The control group receives an introductory educational session only. Primary and secondary outcomes include clinical and behavioral measures, such as HbA1c and weight change, access to and utilization of care (i.e. appointment keeping and use of specialty care), and knowledge and practice of physical activity and healthful eating. Additionally, information regarding CHW characteristics, the processes and mechanisms for influencing healthful behavior change, and fidelity of the intervention are collected. Outcomes are measured at Baseline, 3-Months, 6-Months for both groups, and at 12-Months for the treatment group.

**Discussion:**

To our knowledge, this study represents the first attempt to document the efficacy of T2DM management strategies in the NYC Bangladeshi population. Thus, future qualitative and quantitative findings of the submitted protocol will fill an important gap in the health disparities literature.

**Trial registration:**

NCT02041598

## Background

New York City (NYC) is currently home to the largest Bangladeshi population in the United States (US). From 2000 to 2010, the Bangladeshi population in NYC experienced a 119% increase in size, growing from 28,269 to 61,788 individuals [1]. In contrast, the Asian American community in NYC overall increased by 30%. The addition of approximately 33,000 Bangladeshis to the city’s diverse cultural landscape represented the second-largest numerical increase among Asian groups in the city in the last decade. World Health Organization (WHO) estimates placed Bangladesh on the top ten list of countries with the highest number of estimated cases of Type 2 diabetes mellitus (T2DM) prevalence in 2000 [2]. WHO projections for 2030 estimate the prevalence of T2DM in Bangladesh to increase to over 11 million. Although the prevalence of T2DM among Bangladeshis has been well documented in the home country [2-5], as well as in Canada and the U.K [6,7], little is known about its prevalence and management practices among US Bangladeshis. Studies conducted among South Asian populations (which include individuals from India, Pakistan, and Bangladesh) in the US have found that these groups are 4 to 7 times more likely to suffer from T2DM than the general population [8-10]. Several community-based samples of Bangladeshis in NYC report diabetes prevalence rates in the range of 17-25%, as compared to 10% in non-Hispanic whites [10-12].

In addition to being disproportionally at risk for diabetes, Bangladeshis in NYC have a unique demographic profile that may impact access to and utilization of care, resulting in poorer health outcomes. According to the US Census, 53% of Bangladeshis in NYC speak English less than “very well” and approximately 33% live below the poverty line (compared to the citywide average of 19%) [1]. The above demographic factors, coupled with an increased risk profile for diabetes, highlight the need for culturally- and linguistically-relevant interventions in this community. Community Health Workers (CHWs) have been suggested as a culturally-based strategy to address this need among other ethnic and immigrant populations. CHWs are generally indigenous to the community in which they work -- ethnically, linguistically, socioeconomically, and experientially – providing them with a unique understanding of the norms, attitudes, values, and strengths of community members [13,14]. This understanding is incredibly valuable given the growth of minority and underserved populations whom health care providers have difficulty reaching or communicating with [15,16]. Moreover, given that the nature of chronic disease requires long-term management and life-style modification, the use of CHWs is increasingly being viewed as a low-cost approach to improving community health and well-being, reducing health disparities, and bridging the cultural and social barriers between underserved communities and the health care system [14,17].

While there has been formative work to study the role of health beliefs, behaviors, and barriers to/facilitators of T2DM management in this community [18,19], no known studies to date have tested the efficacy of a CHW intervention in the Bangladeshi community. We present the protocol of a research study designed to fill this gap in knowledge. The DREAM (Diabetes Research, Action, and Education for Minorities) Project is a 6-month randomized controlled trial (RCT) that utilizes the CHW model to improve diabetic control and management among Bangladeshis living in NYC.

## Methods/Design

### Objectives

The primary aim of the current study is to assess the efficacy of a CHW intervention to improve management and control among Bangladeshis with T2DM in NYC. Secondary aims include examining the intervention’s effect on diabetes knowledge, self-management practices, health behaviors, and access to primary and specialty care services among study participants.

### Ethics approval

The study protocol and procedures were approved by the Institutional Review Board at the New York University School of Medicine on July 6, 2009. Written informed consent is obtained from study participants by trained CHWs or study personnel prior to enrollment.

### Sample size calculations

The size of the study population is based on the primary outcome measure of HbA1c and was estimated by using previous changes observed in a pilot study as well as in previous research [19,20]. Control group HbA1c estimates were based on hospital patients receiving usual care. An absolute difference of 0.5% in HbA1c between the groups (6.7 vs. 6.2) with a power of 0.8 and significance level of 0.05 may be detected with a total of 40 individuals in each group, and an absolute difference of 0.3% in HbA1c between the groups (6.7 vs. 6.4) with a power of 0.8 and significance level of 0.05 may be detected with a total of 120 individuals in each group. The sample size calculation was based on a medium effect size of the primary outcomes between the intervention and control group. Approximately 256 enrolled study participants were randomized each to the treatment and control groups. We anticipated that 75% of study participants would be followed to completion of the study at 6 months (n = 96 in each group).

### Study participants

The study population of interest is Bangladeshi individuals residing in NYC that have been diagnosed with T2DM. Study eligibility includes the following:

(a) confirmed clinical diagnosis of T2DM with a Hemoglobin A1c (HbA1c) of ≥ 6.5%; *and*

(b)  male or female between the ages of 21–85 years old; *and*

(c) willingness to be randomized to either treatment or control groups.

Participants are ineligible for enrollment in the study if s/he:

(a) is or was on renal dialysis; *or*

(b)  is experiencing an acute or terminal illness or serious mental illness; *or*

(c)  had a history of recent coronary event within the last 3 months of recruitment; *or*

(d)  is pregnant at the time of recruitment; *or*

(e)  experienced other severe medical conditions that might preclude participation; *or*

(f) has poor short-term prognosis (expected death in <2 years); *or*

(g)  is participating in another research study.

### Study design

Aggregated patient registry data from a large, public hospital in NYC was used to gauge the approximate size of the community served at this particular healthcare institution. A targeted enrollment of n = 256 was used for this study as previously described in the power calculation. The projected sample size also takes into account previous literature detailing refusal and attrition rates for peer- and CHW-led health interventions [21-23].

The study is a two-arm RCT design. Following screening, consenting, and study enrollment, participants are randomly assigned to either treatment or control group by a blinded study staff member using IBM SPSS Statistics, Version 19.0 (IBM Corp). Study groups are stratified by gender and age. Both study staff and participants are notified of subject randomization allocation. Additionally, all participants randomized to the control groups are offered the opportunity to receive the full intervention after serving as a control for the 6-month study period. This strategy is part of a multi-pronged effort to improve data collection rates among participants randomized to the control group, as well as an effort to conduct the study in alignment with the principles of community-based participatory research (CBPR) and in response to community concerns regarding the ethics of withholding potentially beneficial interventions for community members.

### Study recruitment

**Figure 1:**
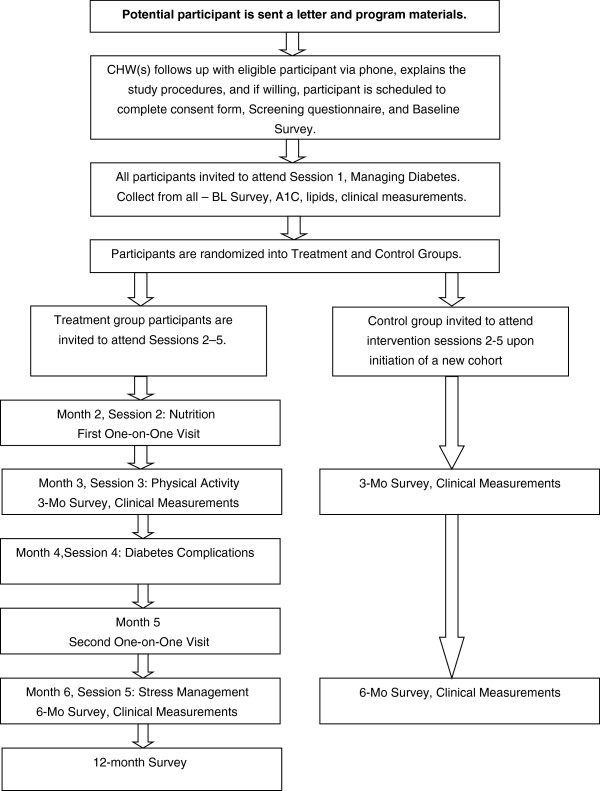
Recruitment process flowchart (Mass Mailing).

We drew upon literature which suggested that a multi-pronged recruitment approach that considers cultural and contextual factors impacting participation in research should be applied [24-27]. One strategy employed includes the use of a mass mailing and phone-follow-up to potential participants recruited from a public hospital in NYC that serves a large Bangladeshi patient population. Patients are sent introductory letters describing the study and providing promotional materials, both of which were in Bengali and English. Patients then receive a follow-up phone call from a CHW approximately two weeks later, at which time additional study information is provided and eligibility information is obtained via a standardized script and screening questionnaire. The screening tool includes questions related to the protocol-specified inclusion/exclusion criteria, as well as questions regarding planned travel to the home country, which was identified as a significant barrier to retention during the formative phase of this study [18]. The above recruitment protocol has also been previously used in several diabetes and lifestyle interventions primarily targeting Caucasian and Hispanic participants [28,29]. Figure 1 illustrates the flow of recruitment and enrollment using this approach.

**Figure 2:**
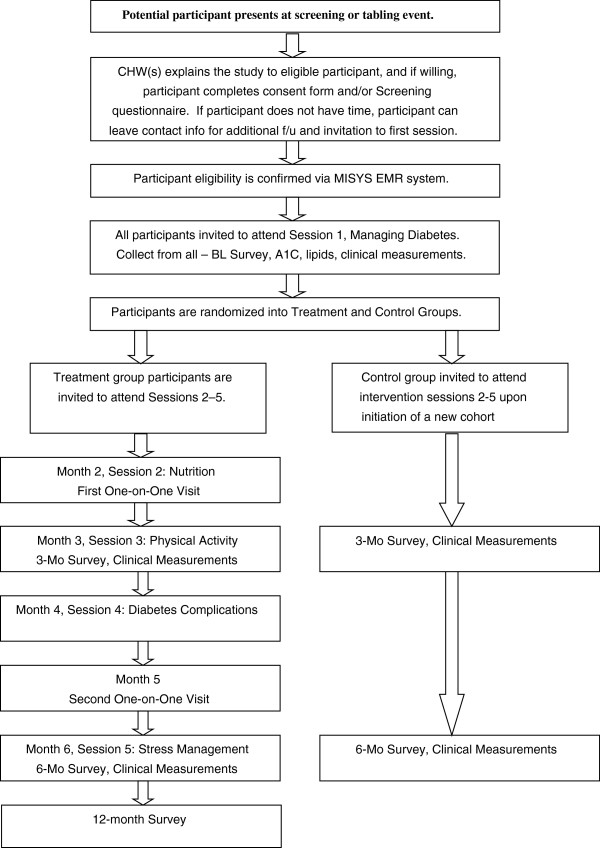
Recruitment process flowchart (Recruitment/Tabling Event).

In addition to the mailing and phone follow-up, screening and tabling events are also held in the hospital atrium 2–3 times per week during the recruitment periods. CHWs provide study recruitment materials in Bengali to interested study participants who are passing to and from clinic appointments, and screen those interested in participation. During these encounters with potential participants, contact information is also obtained, so that CHWs can complete additional follow-up and continue to build rapport with interested participants prior to obtaining informed consent for participation in the intervention. Figure 2 illustrates the flow of recruitment and enrollment using this approach.

Other recruitment methods employed include “snowball” or word-of-mouth referrals, which are accepted from screened and enrolled participants. The project staff also works with local faith-based and community-based organizations to do outreach and screening events in neighborhoods in close proximity to the hospital site. Lastly, articles are placed in local Bangladeshi newspapers which include information regarding the study and (in some cases) contact information for study staff. These articles are published with the intention of promoting general awareness of the program among the community of interest, and not as a direct recruitment tool.

### CHW intervention

Individuals randomized to the treatment group receive a multi-component intervention comprising of group educational sessions, one-on-one visits, and phone follow-up by one of three full-time CHWs. Group educational sessions occur once per month, for five months, and are held in a community-based or hospital setting. Each session is conducted in Bengali and involves a discussion of different content areas related to T2DM, such as nutrition, physical activity, and complications of diabetes. Educational sessions are guided by the Health Belief Model [30] and Social Support Theory [31]. For example, in describing diabetes, CHWs explain the prevalence of T2DM and described risk factors. These discussions are intended to make participants aware of their risk and to enhance participants’ acceptance of their diabetes diagnosis—i.e., altering participants’ perceived susceptibility to diabetes. Educational sessions are also supplemented with materials/handouts that have been adapted from standardized curriculums and translated for use in the Bangladeshi community [19]. Materials are tailored with specific cultural and religious practices in mind, with guidance and input from both project CHWs and a community coalition comprised of representatives from academic institutions, healthcare agencies, and community-based organizations [32]. Project staff and coalition also utilize results from a mixed-methods formative research study that were completed prior to study implementation with the target community to help guide the process of tailoring materials and curriculum content [18].

In addition to educational sessions, treatment group participants receive two one-on-one visits during the six month study period. Each visit provides study participants with the opportunity to discuss individualized care needs related to diabetic management with a CHW, as well as to discuss goal-setting around health behaviors using motivational interviewing techniques. CHWs also help to provide referrals to specialty care (e.g. podiatry and dentistry), as well as to social service or community-based resources for needs related to housing, food assistance, immigration, etc. Lastly, CHWs utilize phone follow-up between educational sessions and one-on-one visits to both encourage adherence to discussed care plans, as well as to improve study retention and data collection rates by minimizing study drop-out. All of the above interactions with study participants are documented in CHW progress notes and call logs to ensure fidelity to study protocol as well as to capture secondary study outcomes related to access to and utilization of care.

Individuals randomized to the control group receive a one-time, introductory educational session only. This session focuses on general concepts regarding diabetes management, and is the same introductory session received by the treatment group. Individuals randomized to the control group are instructed to seek medical care as usual from their regular physician. Previous literature has underscored the importance of providing meaningful incentives as a strategy to maximize retention in community-based studies [33]. Accordingly, participants are provided round-trip MetroCards® ($4.50 - $5 value) at each educational session and at each data collection timepoint. Additionally, treatment group participants are given a small incentive related to the session topic (e.g. bag of brown rice, pill box, pedometer, etc.) at each session, as well as a ticket towards a $100 raffle drawing to be completed at the end of the intervention period.

### Data collection

**Figure 3:**
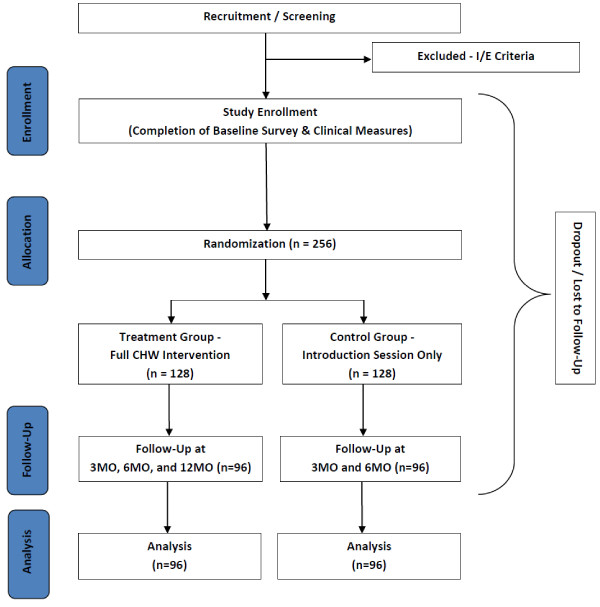
Overall study design flowchart.

Data collection occurs with treatment and control group participants at Baseline, 3-Month, and 6-Month timepoints, with a +/- 30 day window. Additionally, the treatment group is contacted at the 12-Month timepoint to obtain long-term follow-up information. Within the clinical setting, providers collect Hemoglobin A1c (HbA1c) levels and lipid profile according to the standard of care lab schedule for management of each participant. Outside of the clinical setting, questionnaire data and several clinical endpoints (height, weight, blood pressure, and waist/hip circumference) are obtained; all procedures are completed in-language by CHWs or trained study personnel. In addition to the above timepoints, questionnaire data is collected at each one-on-one visit pertaining to access and utilization of primary and specialty care, as well as barriers encountered in maintaining a healthy diet, engaging in physical activity, and managing stressors. Figure 3 demonstrates procedures for each study group and overall flow of study design.

### Study outcomes

Primary study outcomes include: (1) decreased levels of HbA1c, lipid profiles, and systolic and diastolic blood pressure and a greater proportion of individuals with controlled blood pressure; (2) greater access and utilization of healthcare; and (3) a positive impact of knowledge and practice of physical activity and healthful eating. Secondary outcomes include: (1) increased levels of perceived social support; (2) greater perceived benefits and lower perceived barriers; and (3) higher levels of self-efficacy.

Clinical measures included HbA1c, lipid profile (cholesterol, HDL, LDL, and triglycerides), systolic and diastolic blood pressure, height, and weight. BMI is calculated at each timepoint using current weight and height recorded at baseline. Waist-to-hip ratio is calculated using waist and hip circumference measurements obtained at each timepoint. Blood pressure is collected using previously validated methods for community-based settings, given that data collection frequently occurred outside of the hospital [34]. Three resting blood pressure measurements approximately 2–3 minutes apart are collected at each timepoint using an OMRON HEM 711-AC automatic blood pressure monitor with participants in a seated position. The average of the second and third blood pressure readings is recorded as the final value. HbA1c and lipid profile were measured through a laboratory blood test.

#### ***Predisposing & reinforcing characteristics***

Information on predisposing characteristics of study participants is collected at baseline only, and include health access and insurance status, length of time in the US, acculturation, marital status, socioeconomic status, and personal and family history of T2DM. Measures were primarily drawn from the Behavioral Risk Factor Surveillance System (BRFSS) [35]. Information on reinforcing characteristics, including self-efficacy [36], social support and social capital [37], and religiosity are collected at each timepoint.

#### ***Knowledge and health behaviors***

At each time point, knowledge related to T2DM is assessed. Existing measures were adapted to assess diabetes management practices [38], knowledge acquired from each educational session, dietary practices [39], tobacco usage [35], patterns of physical activity [40], medication adherence [41], and mental health [42].

#### ***Knowledge and acceptability of CHWs***

At each follow-up timepoint, we assess the mechanisms through which CHWs operate to fulfill their role as a bridge to the healthcare system and advocates for their community. A measure to assess these mechanisms included items related to trust, communal concordance, honesty, communication, empowerment, and resource linkages. Table 1 outlines all of the above measurement domains & data collection timepoints in more detail.

**Table 1:** Measurement domains & data collection timepoints

**Component**	**Baseline**	**3 MOS**	**6 MOS**	**12 MOS**
**Socio-demographic (pre-disposing characteristics):**				
Sex, age, marital status, country of birth, years in U.S., education, household size, home language, English fluency	✓			
Employment Status	✓	✓	✓	✓
Health insurance	✓			
Barriers to healthcare	✓			
**Reinforcing characteristics:**				
Social support	✓	✓	✓	✓
Self-efficacy	✓	✓	✓	✓
Social capital	✓	✓	✓	✓
Religiosity	✓	✓	✓	✓
**Knowledge and health behaviors:**				
Diabetes knowledge	✓	✓	✓	✓
Diabetes management	✓	✓	✓	✓
Dietary practices	✓	✓	✓	✓
Food behavior	✓	✓	✓	✓
Tobacco use	✓	✓	✓	✓
Physical activity	✓	✓	✓	✓
Medication adherence	✓	✓	✓	✓
Mental health	✓	✓	✓	✓
**Clinical measurements:**				
HbA1c	✓	✓	✓	✓
Blood pressure	✓	✓	✓	✓
Height				✓
Weight	✓	✓	✓	✓
BMI	✓	✓	✓	✓
Waist, Hip, Waist-to-Hip ratio	✓	✓	✓	✓
Lipid profile	✓	✓	✓	✓
**Knowledge and acceptability of CHWs**				
	✓	✓	✓	
**Program evaluation**				
				✓

### Data analyses

Analyses to compare the treatment and control group participants are conducted at baseline to detect significant differences. Student t-tests for normally distributed variables (after necessary transformations) and chi-squared tests for categorical variables were used. In the event that randomization did not control for differences between the treatment and control groups on baseline characteristics, we statistically controlled for those differences in subsequent analyses of program effects. Descriptive statistics are conducted to characterize study participants and to evaluate the distribution of key exposure and outcome variables, as well as to describe the factors that affect utilization of health care services and the risk and protective factors for diabetes. Summary data on medical history, length of time in the US, access and utilization to primary health care services, and social characteristics (e.g., marital status, employment, education) is reported.

The primary goal of this study is to evaluate intervention efficacy, using the generally accepted approach in evaluating efficacy trials, Intention to Treat (ITT) [43]. The analysis for each aim will be comprised of t-tests for continuous variables and chi-square tests for categorical variables. Indexes will be created for scale variables and examined as continuous variables. Mann–Whitney U Tests will be applied for indices with non-parametric distributions.

Each aim will be tested using multivariate logistic regression analysis. The base model will consider the intervention group as the primary predictor variable. Interaction terms between treatment and follow-up time will be developed to assess whether the effect of treatment varied over time. A third set of models included other covariates identified in the preliminary analysis as potential confounders that may reduce the likelihood of confounding bias. Goodness-of-fit of the final models will be assessed using the Hosmer-Lemeshow test.

## Discussion

To our knowledge, this study represents the first attempt to document the efficacy of diabetes management strategies in the NYC Bangladeshi population. Thus, the future qualitative and quantitative findings of the submitted protocol will fill an important gap in the health disparities literature. Additionally, this study aims to characterize the specific mechanisms through which CHWs operate to fulfill their role as a bridge between the community and the healthcare system. This information will help to provide a more concrete exploration of the specific characteristics of a CHW that may be related to their efficacy in improving health outcomes. Lastly, we anticipate that the collection and reporting of detailed process evaluation metrics related to both the study recruitment and implementation phases will help to inform the design of future CHW interventions/programs.

There are several limitations to the current study. First, blinding study staff or participants to randomization allocation is not feasible, and thus may result in ascertainment bias and subsequent threat to study validity. Additionally, since control group participants do receive an introductory session that contains general information regarding diabetes management, individuals may make subsequent behavior changes leading to positive clinical outcomes that would make observation of between-group differences problematic. However, because the study is guided by CPBR principles, investigators felt that the current study design was a reasonable tradeoff between what is scientifically rigorous and what is valuable to the community. Lastly, since many individuals reside in the same neighborhoods in NYC, there is a potential for contamination bias between the two arms to occur. Investigators will attempt to control for this through measuring interaction between study participants via the survey questionnaire.

Recruitment for the study began in February 2011, and will be conducted in phases or cohorts (rather than on an ongoing basis). Enrollment and data collection is scheduled to conclude by November 2014, at which time final data analysis will commence and study findings will be reported in a separate publication.

## Abbreviations

BMI: Body mass index; BRFSS: Behavioral risk factor surveillance system; CBPR: Community-based participatory research; CHW: Community health worker; HbA1c: Hemoglobin A1c; NYC: New York City; T2DM: Type 2 diabetes mellitus; UK: United Kingdom; US: United States.

## Competing interests

The authors declare that they have no competing interests.

## Authors’ contributions

All authors contributed substantially to the manuscript and approved this submission. LR and NI supervised the implementation of the CHW intervention and took the lead on writing of the manuscript. LW created the study databases and contributed to the writing on sections of the manuscript related to study analysis. SDT, MT, RM, MR, and CTS edited the paper and contributed to overall study design and implementation. NI bears overall responsibility for the design, ethnical conduct, and publication of the study.

## Pre-publication history

The pre-publication history for this paper can be accessed here:

http://www.biomedcentral.com/1471-2458/14/177/prepub
